# Revealing Time-Unlocked Brain Activity from MEG Measurements by Common Waveform Estimation

**DOI:** 10.1371/journal.pone.0098014

**Published:** 2014-05-30

**Authors:** Yusuke Takeda, Kentaro Yamanaka, Noriko Yamagishi, Masa-aki Sato

**Affiliations:** 1 Department of Computational Brain Imaging, ATR Neural Information Analysis Laboratories, Kyoto, Japan; 2 Graduate School of Human Life Sciences, Showa Women’s University, Tokyo, Japan; 3 Department of Cognitive Neuroscience, ATR Cognitive Mechanisms Laboratories, Kyoto, Japan; 4 Brain Networks and Communication Laboratory, Center for Information and Neural Networks, National Institute of Information and Communications Technology, Osaka, Japan; 5 Japan Science and Technology Agency, PRESTO, Saitama, Japan; University College of London - Institute of Neurology, United Kingdom

## Abstract

Brain activities related to cognitive functions, such as attention, occur with unknown and variable delays after stimulus onsets. Recently, we proposed a method (Common Waveform Estimation, CWE) that could extract such brain activities from magnetoencephalography (MEG) or electroencephalography (EEG) measurements. CWE estimates spatiotemporal MEG/EEG patterns occurring with unknown and variable delays, referred to here as unlocked waveforms, without hypotheses about their shapes. The purpose of this study is to demonstrate the usefulness of CWE for cognitive neuroscience. For this purpose, we show procedures to estimate unlocked waveforms using CWE and to examine their role. We applied CWE to the MEG epochs during Go trials of a visual Go/NoGo task. This revealed unlocked waveforms with interesting properties, specifically large alpha oscillations around the temporal areas. To examine the role of the unlocked waveform, we attempted to estimate the strength of the brain activity of the unlocked waveform in various conditions. We made a spatial filter to extract the component reflecting the brain activity of the unlocked waveform, applied this spatial filter to MEG data under different conditions (a passive viewing, a simple reaction time, and Go/NoGo tasks), and calculated the powers of the extracted components. Comparing the powers across these conditions suggests that the unlocked waveforms may reflect the inhibition of the task-irrelevant activities in the temporal regions while the subject attends to the visual stimulus. Our results demonstrate that CWE is a potential tool for revealing new findings of cognitive brain functions without any hypothesis in advance.

## Introduction

Brain activities related to cognitive functions, such as attention and decision making, occur with unknown and variable delays after stimulus onsets. Therefore, to reveal such brain activities from magnetoencephalography (MEG) or electroencephalography (EEG) measurements, it is necessary to examine spatiotemporal MEG/EEG patterns occurring with unknown and variable delays after stimulus onsets, which are referred to as unlocked waveforms in this paper. There are two difficulties in examining unlocked waveforms: their estimation and their interpretation.

The conventional averaging procedures, such as stimulus-triggered averaging, cannot be used to estimate unlocked waveforms because they cancel out the unlocked waveforms owing to their variable delays. Previously, several methods have been proposed for examining unlocked brain activities, such as using time-frequency analyses [1]–[3]. To avoid the cancellation of unlocked activities, time-frequency analyses are first applied to single-trial EEG epochs, and then the obtained time-frequency powers are averaged across trials. The limitation of this method is that it only provides power information on the unlocked activities, while some information is lost, such as phase relations across channels, frequencies and time. To preserve the rich information of unlocked activities, it is preferable to estimate their waveforms. Some studies have proposed methods to estimate waveforms of unlocked activities [4]–[6]. However, these methods have a limitation in that they assume only one waveform in a MEG/EEG epoch and cannot estimate multiple waveforms. In the case of cognitive stimulus-response tasks, such as Go/NoGo tasks, it is assumed that there are three types of waveforms: stimulus-locked, response-locked, and unlocked. Therefore, to estimate unlocked waveforms from MEG/EEG data during cognitive tasks, estimating multiple waveforms is necessary. Recently, we proposed a general method to estimate MEG/EEG waveforms that are common across trials (Common Waveform Estimation, CWE) [7]. The main advantage of CWE is its ability to estimate multiple waveforms without hypotheses about their shapes, even if their delays are variable and unknown. Therefore, applying CWE is a suitable solution for the estimation of unlocked waveforms in studies of cognitive functions.

However, the second difficulty, interpretation of the estimated unlocked waveforms, remains to be solved. Many studies have already examined the roles of MEG/EEG waveforms by comparing their amplitudes across different conditions. For example, the role of P300 was examined by comparing amplitudes across target and non-target conditions. If the amplitude is larger in one condition that needs a specific brain function, such as attention, the waveforms are believed to reflect this. In this procedure, it is implicitly assumed that compared waveforms reflect the same brain function based on the fact that they occur with the same delay to the same event, such as stimulus onsets, in similar conditions. If waveforms in different conditions reflect different brain functions, such as attention and decision making, comparing their amplitudes does not make sense. Because they are not time-locked to externally observable events, it is not clear whether unlocked waveforms estimated from different conditions reflect the same brain function. Therefore, comparing amplitudes of unlocked waveforms may result in a misleading interpretation. This difficulty of interpretation discourages the use of CWE for examining the cognitive brain activities occurring with unknown and variable delays.

The purpose of this study is to demonstrate the usefulness of CWE in cognitive neuroscience studies. Toward achieving this aim, we show the procedure used to examine the role of the unlocked waveform. In this procedure, we attempted to estimate the strength of the brain activity of the unlocked waveform under various conditions. We made a spatial filter to extract the component that reflects the target brain activity corresponding to the unlocked waveform. This spatial filter was applied to MEG data under various conditions. The powers of the components extracted by the spatial filter were calculated while assuming that these powers indicate the strengths of the target brain activity.

This study was conducted in a data-driven way. MEG epochs during the Go trials of a visual Go/NoGo task were assumed to consist of stimulus-locked, response-locked, and unlocked waveforms. Without any hypotheses on their shapes, using CWE allowed us to estimate the three waveforms from the MEG epochs. We found that the estimated unlocked waveforms had large alpha oscillations at 8–10 Hz around the temporal areas. Together with the suggested roles of alpha oscillations in previous studies [8]–[13], we hypothesized that the unlocked waveforms reflected the inhibition of the task-irrelevant activities in the temporal regions while the subject attends to the visual stimulus. We calculated the powers of the components reflecting the brain activities of the unlocked waveforms under different conditions: a passive viewing, a simple reaction time, and Go/NoGo tasks. Using these powers, we tested our hypothesis in the following three ways. First, to examine whether the unlocked waveforms are related to attention, we compared the powers across conditions that either require or do not require attention. Second, to examine whether the unlocked waveforms reflect the inhibition of the task-irrelevant activities, we examined the relation between the powers and the variability of reaction times (RTs) under the Go condition. Finally, to examine whether the unlocked waveforms are specifically related to “visual” attention, we compared the powers between the “visual” and “auditory” Go/NoGo conditions. The above analyses all supported our hypothesis.

## Materials and Methods

### Subjects

Nine healthy subjects participated in this study. All gave written informed consent for the experimental procedures, which were approved by the ATR Human Subject Review Committee. All had normal or corrected-to-normal visual and auditory acuity. One subject was excluded owing to too many wrong responses (about 30%) in the Go trials of the Go/NoGo task. All analyses were conducted using the remaining eight subjects (ages 32.1±7.1 (mean ± standard deviation [SD]), including one female).

### Experimental Design

We conducted MEG and functional Magnetic Resonance Imaging (fMRI) experiments. The fMRI data were used as prior information in estimating the current sources of the MEG waveforms [14].

**Figure 1:**
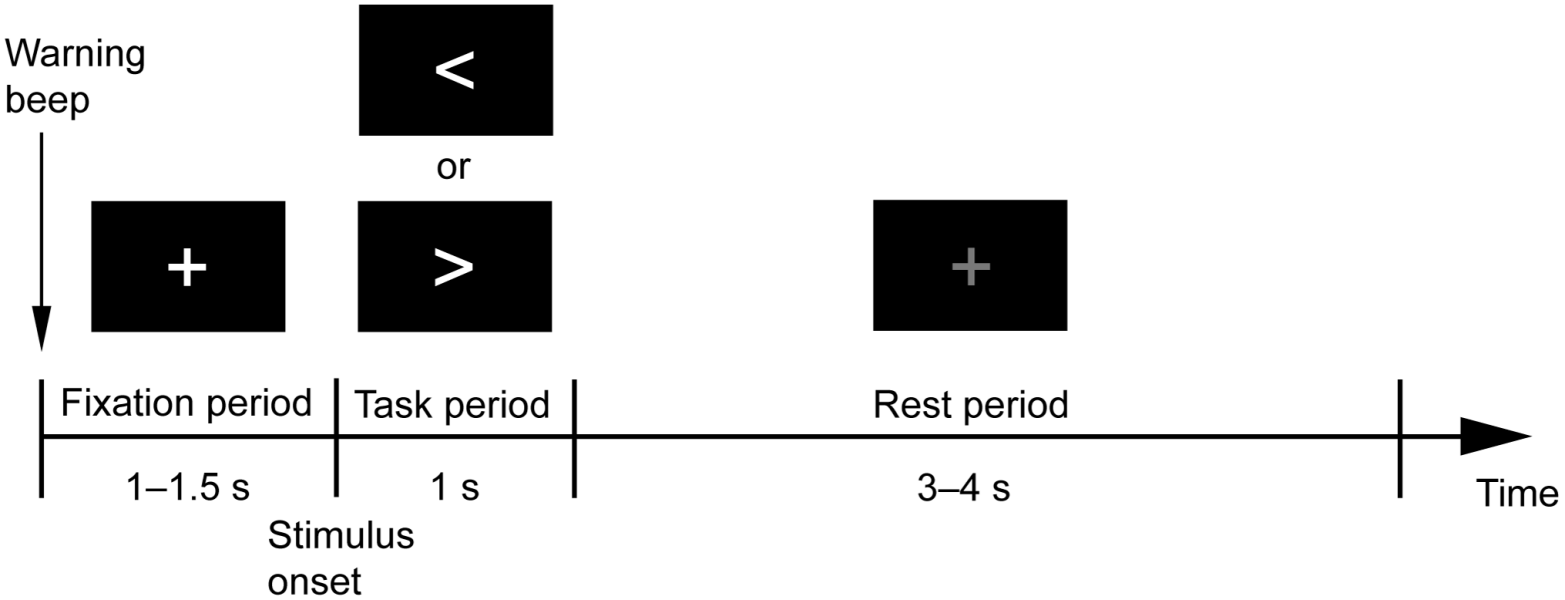
Experimental design. Each trial began with a warning beep. Following the beep, a white cross was presented for a variable duration of 1–1.5 s. Subjects were instructed to fixate on it. Then a cue stimulus, “>” or “<”, was presented for 1 s to instruct subjects to initiate the task. Following the cue stimulus, a gray cross was presented for a variable duration of 3–4 s. Subjects were allowed to blink only during this period.

For the MEG and fMRI experiments, the same experimental stimuli and event-related design were used (Figure 1). Each trial began with a warning beep. Following the beep, a white cross was presented for a variable duration of 1–1.5 s, and subjects were instructed to fixate on it. Then a cue stimulus “>” or “<” was presented for 1 s to instruct subjects to initiate a task. Cue stimuli “>” and “<” were presented in random order at equal probability. The cue stimulus onset is referred to as the stimulus onset. Following the cue stimulus, a gray cross was presented for a variable duration of 3–4 s. Subjects were allowed to blink only during this period. Each run consisted of 50 trials. Different runs had different sequences.

Three tasks were conducted in the following order: a passive viewing (Passive) task, a simple reaction time (SRT) task, and a Go/NoGo task. In the Passive task, subjects passively viewed the cue stimulus. In the SRT task, subjects pushed a button with their right index finger immediately after the stimulus onset. In the Go/NoGo task, subjects pushed a button with their right index finger immediately after the “>” stimulus onset (Go) and did not push it after the “<” stimulus onset (NoGo). We set the Go condition as the target, in which unlocked waveforms were examined, and used the other conditions for comparison. For the Passive and SRT tasks, all subjects participated in two runs (100 trials). For the Go/NoGo task, one subject participated in five runs (125 Go and NoGo trials) and the other subjects participated in four runs (100 Go and NoGo trials).

### Data Acquisition

#### MEG recording

A whole-head 400-channel system (210-channel Axial Gradiometer and 190-channel Planar Gradiometer; PQ1400RM; Yokogawa Electric Co., Japan) was used for the MEG recording. The sampling frequency was 1 kHz. An electro-oculogram (EOG) value was simultaneously recorded. Before the MEG experiment, the subject’s face and head shape were scanned using a hand-held laser scanner and a stylus marker (FastSCAN Cobra; Polhemus, U.S.A.) for later co-registration of the MEG and MRI results. To measure the head position in the MEG system, four calibration coils were bilaterally mounted on the subject’s temporal skin (two for the superior superciliary and two for anterior subauricular regions). Electromagnetic calibration of the coil positions was conducted before and after each MEG recording run by applying alternating currents to the coils.

#### MRI recording

Three Tesla MR scanners (MAGNETOM Trio 3T for three subjects and MAGNETOM Verio 3T for the others; Siemens, Germany) were used to obtain the structural and functional MRI data.

The following are the acquisition parameters for the T1-weighted images: repetition time 2250 ms, time of echo 3.06 ms, flip angle 9°, slice thickness 1 mm, field of view 256
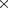
256 mm, imaging matrix 256
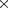
256 and 192 slices.

The following are the acquisition parameters for the echo-planar images (EPIs): repetition time 3 s, time of echo 30 ms, flip angle 80°, slice thickness 3 mm, field of view 192
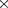
192 mm, and imaging matrix 64
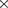
64 with 50 slices for the three subjects and 47 slices for the others.

### Data Analysis

#### MEG data

Preprocessing. To remove artifacts and noise from the MEG data and reshape them for the common waveform estimation, the MEG data were passed through a low-pass finite impulse response (FIR) filter with a cutoff frequency of 40 Hz, sampled at 100 Hz, and passed through a high-pass FIR filter with a cutoff frequency of 2 Hz. Using the reference sensor data, environmental noise was removed by time-shift Principal Component Analysis (PCA) [15]. The MEG data were segmented into 5-s epochs from 2 s before the stimulus onset to 3 s after it. For each sensor, EOG artifacts were removed by generating a multiple linear regression model to predict eye-movement-related components in the MEG epochs by the EOG data and then removing the prediction from the MEG epochs. RTs were defined as the intervals between the stimulus onsets and the button-push signal (response) onsets. We excluded the trials in the SRT task and the Go condition with RTs either shorter than 0.1 s or longer than 0.8 s. Trials were also excluded if subjects made button-push responses in the Passive task and the NoGo condition. If the maximum value of the MEG epoch from 0.5 s before the stimulus onset to 1 s after it exceeded 2
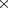
10^−12^ T absolute value, we excluded that trial. Cardiac artifacts and sensor noise were removed by Independent Component Analysis (ICA) [16] using MATLAB code provided by Makeig et al. [17]. We obtained 99.9±0.4 trials for the Passive task, 97.4±4.3 trials for the SRT task, 100.6±9.7 trials for the Go condition, and 98.1±3.9 trials for the NoGo condition. The mean RTs for the SRT task and Go conditions were 0.29±0.05 s and 0.38±0.04 s, respectively.

Common Waveform Estimation. To estimate unlocked waveforms, we applied CWE to the preprocessed 400-channel MEG epochs of the Go condition.

CWE is a general method for estimating waveforms that are common across trials from MEG/EEG epochs. When waveforms spatiotemporally overlap, the averaging procedure cannot estimate exact waveforms because they are mutually contaminated [18]–[23]. Furthermore, when the delays of waveforms are variable and unknown, the averaging procedure cannot be used [3], [24]. CWE was proposed to overcome these limitations and provide a way to work in more general and severe situations, where we do not know the number of waveforms common across trials, their delays in individual trials, and all of the waveforms. In CWE, a MEG epoch of a channel is assumed to consist of waveforms common across trials and is expressed by
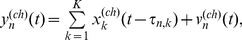
(1)


where 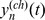 represents a MEG epoch of channel 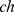 in trial 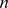, 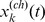 represents the 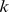-th waveform of channel 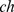, 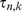 represents the delay of 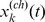 in trial 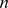, 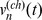 represents the noise of channel 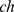 in trial 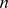, and 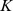 represents the number of waveforms. Only from 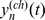, CWE estimate 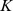, 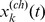, and 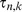. In the Fourier domain, they are iteratively searched for, with the aim of minimizing the residual error between the observed and reconstructed MEG epochs. Using CWE, we can obtain exact waveforms, which are not contaminated with each other, without any knowledge of their shapes and delays in individual trials.

In this experimental design, we assumed three waveforms in a MEG epoch: a stimulus-locked waveform, a response-locked waveform, and an unlocked waveform. We set the delays of the stimulus-locked waveforms to 0 s and the delays of the response-locked waveforms to the RTs. That is, we simplified the assumption from Eq. (1) to
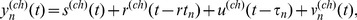
(2)


where 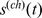 represents the stimulus-locked waveform of channel 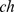, 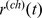 represents the response-locked waveform of channel 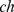, 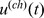 represents the unlocked waveform of channel 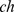, 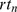 represents the RT in trial 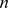, and 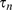 represents the delay of the unlocked waveform in trial 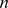. By CWE, we estimated 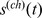, 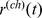, 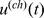, and 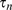 from 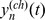 and 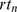. We searched for the delays of the unlocked waveforms by setting the initial delays to Gaussian random numbers (0.35±0.20 s) and the range of the delay to ±0.25 s. After the search, it was necessary to adjust the averages across the trials of the delays. This is because the delays are defined as the interval between the stimulus onsets and the onsets of the unlocked waveforms, and the onsets of the unlocked waveforms are arbitrarily determined when searching for the delays. We adjusted them so that the delays represented the interval between the stimulus onsets and the maximum peak of the unlocked waveforms.

Comparing Estimated Waveforms with Averages across Trials. To examine the validity of the estimation, the estimated stimulus-locked waveforms, response-locked waveforms, and unlocked waveforms were compared with the averages across the trials of the MEG epochs that were triggered on the stimulus onsets (stimulus-triggered averages), the response onsets (response-triggered averages), and the estimated delays of the unlocked waveforms (estimated delay-triggered averages), respectively.

**Figure 2:**
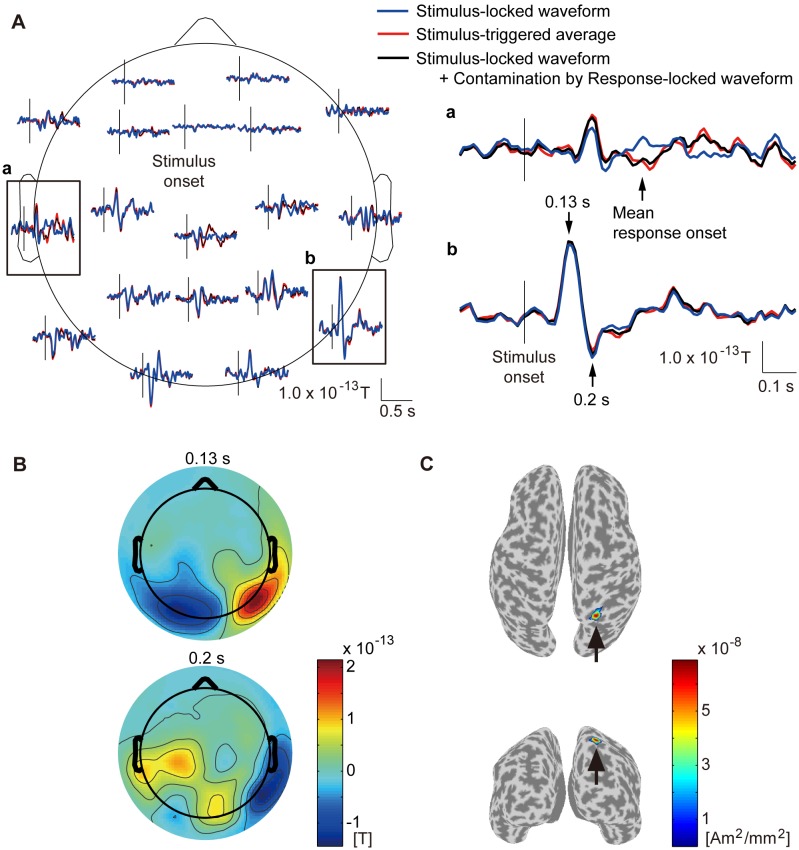
Stimulus-locked waveform (subject 8). **A**: Time course of stimulus-locked waveform. Blue and red lines represent stimulus-locked waveform and stimulus-triggered average. Black lines represent stimulus-locked waveform contaminated by response-locked waveform. Vertical lines represent stimulus onset. Framed plots are enlarged on the right (**a** and **b**). **B**: Spatial patterns of stimulus-locked waveform at 0.13 s (upper) and 0.2 s (lower) after stimulus onset. **C**: Current sources estimated from stimulus-locked waveform. Powers averaged across time from 0 to 0.5 s after stimulus onset are shown. Other subjects show similar results.

**Figure 3:**
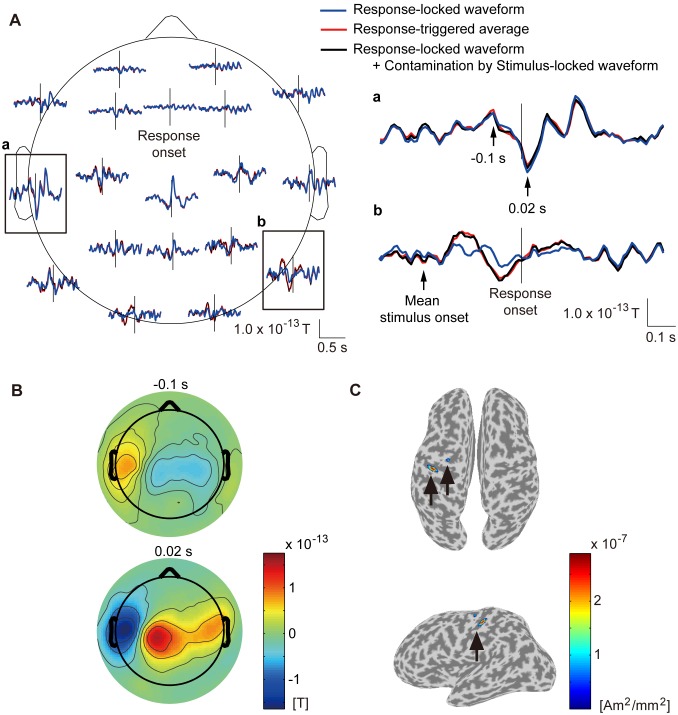
Response-locked waveform (subject 8). **A**: Time course of response-locked waveform. Blue and red lines represent response-locked waveform and response-triggered average. Black lines represent response-locked waveform contaminated by stimulus-locked waveform. Vertical lines represent response onset. Framed plots are enlarged on the right (**a** and **b**). **B**: Spatial patterns of response-locked waveform at −0.1 s (upper) and 0.02 s (lower) after response onset. **C**: Current sources estimated from response-locked waveform. Powers averaged across time from −0.25 s to 0.25 s after response onset are shown. Other subjects show similar results.

Theoretically, the stimulus-triggered average should be more or less different from the stimulus-locked waveform. According to Eq. (2), the stimulus-triggered average is expressed by
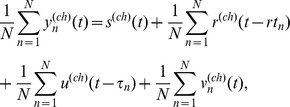



where 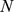 represents the number of trials. This includes not only the stimulus-locked waveform but also contamination by the other waveforms (the second and third terms on the right side). This is also true for the response-triggered average. Indeed, the stimulus/response-locked waveforms are different from the stimulus/response-triggered averages to some extent (see Figures 2A and 3A). The differences are attributed to contamination by the response/stimulus-locked waveforms in the stimulus/response-triggered averages [18]–[23]. To confirm this assumption, we simulated the contaminated waveforms and compared them with the stimulus/response-triggered averages. Stimulus-locked waveforms contaminated by response-locked waveforms were calculated by 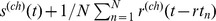. Response-locked waveforms contaminated by stimulus-locked waveforms were calculated by 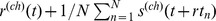.

Current Source Estimation of Waveforms. To examine the current sources of the estimated waveforms, we applied Variational Bayesian Multimodal EncephaloGraphy (VBMEG) [14] to the estimated waveforms of the 210 axial sensors.

From MEG data, VBMEG estimates distributed currents using fMRI data as the prior information on the current variance distribution. The reliability of VBMEG has been confirmed by both computer simulation [14], [25] and experiment [26]–[32].

For each subject, a polygon model of the cortical surface was constructed based on MR structural images using FreeSurfer software [33]. We assumed about 3,000 single-current dipoles equidistantly distributed on and perpendicular to the cortical surface. The fMRI information from the Go condition was used for the prior information on the current variance distribution. The variance magnification parameter was set at 100 [26]. The confidence parameter was set at 10 [26]. A spatial smoothness constraint on the current distribution along the cortical surface was incorporated into the estimation (6 mm full-width at half-maximum). We assumed that the pattern of the cortical activity changes with time. Therefore, we divided the estimated waveforms into time windows (100-ms long with 50-ms overlap). Then, we separately calculated an inverse filter for each time window. Cortical currents were estimated every 10 ms (100 Hz) from the estimated waveforms using the filter. In the overlap periods, they were averaged between two time windows.

Here, we show the power distribution over the cortex of the estimated currents. The power was obtained by calculating 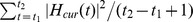, where 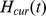 is the Hilbert transform of the estimated current at a dipole, and 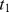 and 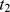 are, respectively, 0 s and 0.5 s after the stimulus onset for the stimulus-locked waveforms, −0.25 s and 0.25 s after the response onset for the response-locked waveforms, and −0.25 s and 0.25 s after the peak for the unlocked waveforms. Powers over 0.3 of the maximum value across the dipoles are shown.

**Figure 4:**
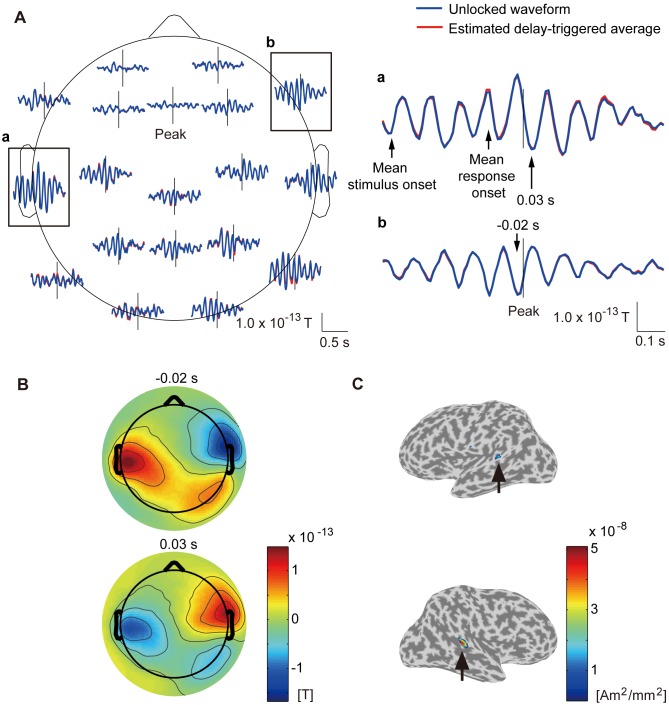
Unlocked waveform (subject 8). **A**: Time course of unlocked waveform. Blue and red lines represent unlocked waveform and estimated delay-triggered average. Vertical lines represent peak time of unlocked waveform. Framed plots are enlarged on the right (**a** and **b**). **B**: Spatial patterns of unlocked waveform at −0.02 s (upper) and 0.03 s (lower) after peak of unlocked waveform. **C**: Current sources estimated from unlocked waveform. Powers averaged across time from −0.25 s to 0.25 s after peak of unlocked waveform are shown. Other subjects show similar results.

Relation between Unlocked Waveform and Alpha Oscillations. The unlocked waveforms have alpha oscillations at 8–10 Hz (see Figure 4A). Therefore, we examined the relation between the unlocked waveform and alpha oscillations originally included in the MEG epochs during the Go condition.

The alpha oscillations in the MEG epochs were extracted by filtering the MEG epochs with a FIR bandpass filter of 8–10 Hz and then aligning the filtered MEG epochs to the estimated delays of the unlocked waveform.

We compared amplitude distributions over space between the unlocked waveform and the alpha oscillations of the original MEG epochs. The amplitude of the unlocked waveform was calculated by
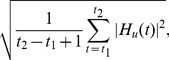
(3)


where 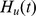 is the Hilbert transform of the unlocked waveform at a sensor, and 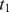 and 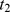 are −0.5 s and 0.5 s, respectively, after the peak of the unlocked waveform. The amplitude of the alpha oscillations in the MEG epochs was calculated by 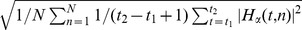, where 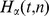 is the Hilbert transform of the alpha oscillation in the MEG epoch at a sensor in trial 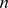, and 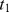 and 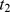 are −0.5 s and 0.5 s, respectively, after the peak of the unlocked waveform.

**Figure 5:**
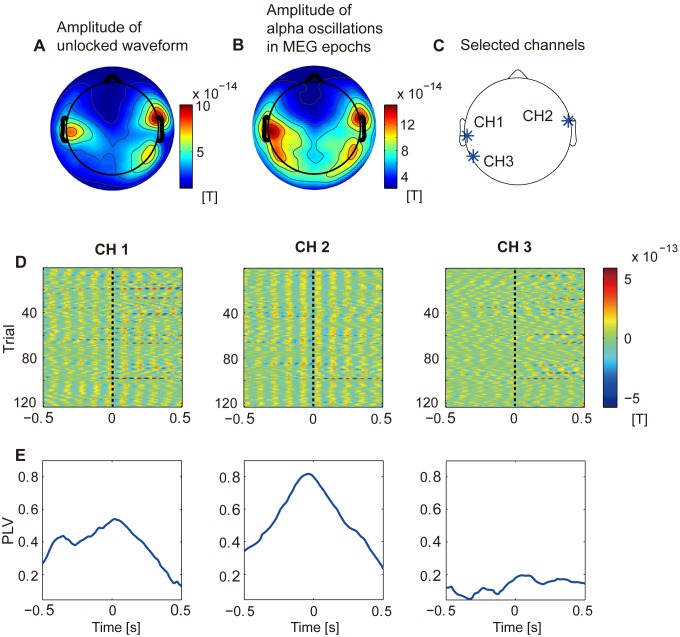
Relation between unlocked waveform and alpha oscillations in MEG epochs during Go condition (subject 8). **A**: Amplitude distribution over space of unlocked waveform. **B**: Amplitude distribution over space of alpha oscillations in MEG epochs. **C**: Selected channels: CHs 1–3. At CHs 1 and 2, unlocked waveform and alpha oscillations in MEG epochs have large amplitudes in common. At CH 3, alpha oscillations in MEG epochs have large amplitudes but unlocked waveform does not. **D**: Time-trial images of alpha oscillations in MEG epochs at CHs 1–3. Vertical dotted lines represent peak time of unlocked waveform. **E**: PLVs at CHs 1–3. In **D** and **E**, time 0 corresponds to peak of unlocked waveform. Other subjects show similar results.

To focus on the similarity and difference between the two amplitude distributions over space (Figure 5A and B), we selected three channels (CHs 1–3). At CHs 1 and 2, the unlocked waveform and the alpha oscillations in the MEG epochs have large amplitudes in common. At CH 3, the alpha oscillations in the MEG epochs have large amplitudes but the unlocked waveform does not have these.

To show the reasons for the similarity and difference visually, we prepared time-trial images [16] for CHs 1–3. In these images, the alpha oscillations in the MEG epochs are shown as color-coded horizontal lines.

To confirm the reasons for the similarity and difference quantitatively, we calculated phase-locking values (PLVs) for CHs 1–3. The PLV was defined as 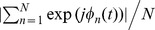, where 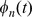 is the angle of the Hilbert transform of the alpha oscillation in the MEG epochs in trial 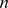 [34].

Properties of Unlocked Waveforms. We examined the properties of the unlocked waveforms in the following three ways.

First, we examined the amplitude distributions over space of the unlocked waveforms. The amplitude was calculated by Eq. (3), where 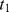 and 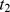 are −0.5 s and 0.5 s, respectively, after the peak of the unlocked waveform. The similarity of the amplitude distributions across the subjects was quantified by calculating the correlation coefficient. For each subject, we calculated the correlation coefficient between his/her own and the mean of the others’ amplitude distributions. A two-tailed sign test was conducted to assess the null hypothesis that the medium value of the correlation coefficients was 0.

Second, we examined the power spectra of the unlocked waveforms. The power spectrum was obtained by taking the discrete Fourier transform of 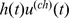, where 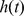 is the Hanning window.

Finally, we examined the correlations between the RTs of the Go condition and the estimated delays of the unlocked waveforms. For each subject, the correlation coefficient between the RTs and the estimated delays of the unlocked waveform was calculated.

Role of Unlocked Waveform. We examined the role of the unlocked waveform. The roles of MEG/EEG waveforms, such as P300, have been examined by comparing their amplitudes across different conditions, implicitly assuming that the compared waveforms reflect the same brain function. However, this procedure cannot be used for unlocked waveforms because unlocked waveforms estimated from different conditions may not reflect the same brain function.

In this study, to examine the role of the unlocked waveform, we attempted to estimate the strength of the unlocked waveform’s brain activity occurring under the various conditions. Using the Denoising Source Separation (DSS) framework [35], [36], we constructed a spatial filter to extract the component reflecting the brain activity of the unlocked waveform. In the DSS framework, spatial filters for separating desired sources can be constructed using prior knowledge about the sources. In this case, the brain activity reflected by the unlocked waveform was regarded as the source. The latencies of the source, which are the estimated delays of the unlocked waveform, were regarded as prior knowledge of the source. The spatial filter to separate the source was constructed using the prior knowledge as follows.

1. A data matrix of size 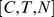 (channeltimetrial) during the Go condition was prepared.

2. The data matrix was reorganized by concatenating the trials along the time dimension to obtain a matrix of size 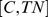.

3. Using PCA, the reorganized data matrix was whitened, i.e. the variance for each channel was normalized, and a whitening matrix 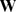 was obtained.

4. The whitened data matrix was reorganized again by trials to form a 3D matrix of size 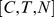.

5. By CWE, the reorganized matrix was decomposed into three 2D matrices of size 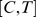: stimulus-locked, response-locked, and estimated delay-locked.

6. A second PCA was performed on the third decomposed matrix, i.e. the estimated delay-locked matrix, and a rotation matrix 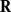 was obtained.

7. The first column of 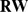, which corresponds to the maximum variance component, was used as the spatial filter.

The above procedure finds the spatial filter to extract the component (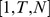), which includes the large temporal pattern (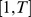) time-locked to the delays of the unlocked waveform. The variance of the temporal pattern over time is maximum under the condition that the variance of the component over time and trials is one. Therefore, the spatial filter enhances the brain activity that occurred at the estimated delays of the unlocked waveform in the same manner across the trials, i.e. the brain activity of the unlocked waveform. By applying the spatial filter to the MEG data of the various conditions, the brain activity of the unlocked waveform can be extracted from the various conditions. We compared the powers of the extracted component across the conditions, assuming that the powers indicate the strengths of the brain activity reflected by the unlocked waveform.

The time course of the power was calculated by taking the moving average of 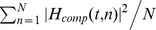, where the window width of the moving average is 0.2 s and 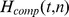 is the Hilbert transform of the component in trial 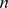. To compare the powers across the conditions, we obtained relative powers by calculating 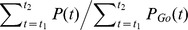, where 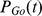, 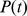 are respectively the time courses of the powers of the Go condition and another condition, and 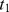 and 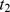 are 0 s and 1 s, respectively, after the stimulus onset. The relative power is >1 if the power of the component of a condition is larger than that of the Go condition, and <1 if it is smaller. A two-tailed sign test was conducted to assess the null hypothesis that the medium value of the relative powers was 1 with Bonferroni correction.

To examine the relation between the powers and the variability of the RTs in the Go condition, for each subject we obtained normalized powers by calculating 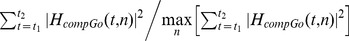, where 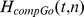 is the Hilbert transform of the component of the Go condition in trial 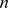, 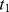 and 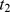 are 0 s and 0.5 s, respectively, after the stimulus onset, and 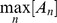 is the maximum value of 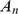 across 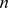. According to the normalized powers, the trials in the Go condition were divided into two groups: smaller power group and larger power group. For each group and subject, the SD of the RTs was calculated. A one-tailed Wilcoxon signed-ranks test was conducted to assess the null hypothesis that the SDs of the two groups did not differ.

#### fMRI data

To obtain the prior information used in estimating the current sources from the estimated waveforms [14], we analyzed the fMRI data during the Go condition by SPM8 (Welcome Department of Cognitive Neurology, UK).

The head motion and slice-timing were corrected, and the images were smoothed using an 8-mm full-width at half-maximum (FWHM) Gaussian filter. The time series of all voxels were high-pass filtered to 1/128 Hz.

Statistical analyses were performed for each subject in the event-related design. The time course of the events, which is the stimulus onset, was convolved with a canonical hemodynamic response function to yield regressors in a general linear model. A parameter was estimated for each regressor, and a t-value of the parameter was calculated for each voxel. The resulting t-values were used for the prior information on the current variance distribution [14].

## Results

### Estimated Waveforms

By using CWE, three types of waveforms were estimated from the MEG epochs of the Go condition: stimulus-locked, response-locked, and unlocked. First, we show the three waveforms. Then, we show the relation between the unlocked waveform and the alpha oscillations originally included in the MEG epochs during the Go condition. Finally, we show the properties of the unlocked waveforms.

#### Stimulus-locked waveform

Figure 2A shows the stimulus-locked waveform estimated by CWE (blue lines) at 19 axial sensors. For comparison, we also show the stimulus-triggered average, which was obtained by averaging the MEG epochs triggered on the stimulus onsets (red lines). Around the occipital areas, the stimulus-locked waveform has as large an amplitude as the stimulus-triggered average (Figure 2Ab, blue and red lines). Around the central and left temporal areas, where the response-locked waveform is large (Figure 3A, blue lines), and around the mean response onset, the stimulus-locked waveform is different from the stimulus-triggered average (Figure 2Aa, blue and red lines). This suggests that the difference is due to the contamination by the response-locked waveform in the stimulus-triggered average [18]–[23]. To confirm this conjecture, the stimulus-locked waveform contaminated by the response-locked waveform was calculated (Figure 2A, black lines). This made the difference disappear (Figure 2Aa, black and red lines). Therefore, CWE successfully separated the stimulus-locked waveform from the contamination of the response-locked waveform, while the stimulus-triggered averaging procedure did not.

Figure 2B shows the spatial patterns of the stimulus-locked waveform at 0.13 and 0.2 s after the stimulus onset. The stimulus-locked waveform is large around the occipital areas.

Figure 2C shows the powers of the current sources estimated from the stimulus-locked waveform. The currents have large amplitudes at the right superior occipital gyrus (indicated by arrows).

#### Response-locked waveform

Figure 3A shows the response-locked waveform estimated by CWE (blue lines) at the 19 axial sensors. For comparison, we also show the response-triggered average, which was obtained by averaging the MEG epochs triggered on the response onsets (red lines). Around the central and left temporal areas, the response-locked waveform has as large an amplitude as the response-triggered average (Figure 3Aa, blue and red lines). Around the occipital areas, where the stimulus-locked waveform is large (Figure 2A, blue lines), and before the response onset, the response-locked waveform is different from the response-triggered average (Figure 3Ab, blue and red lines). This suggests that the difference is due to the contamination by the stimulus-locked waveform in the response-triggered average [18]–[23]. To confirm this conjecture, the response-locked waveform contaminated by the stimulus-locked waveform was calculated (Figure 3A, black lines). This made the difference disappear (Figure 3Ab, black and red lines). Therefore, CWE successfully separated the response-locked waveform from the contamination of the stimulus-locked waveform, while the response-triggered averaging procedure did not.

Figure 3B shows the spatial patterns of the response-locked waveform at −0.1 and 0.02 after the response onset. The response-locked waveform is large around the central and left temporal areas.

Figure 3C shows the powers of the current sources estimated from the response-locked waveform. The currents have large amplitudes at the left precentral gyrus and the central sulcus (indicated by arrows). This is believed to reflect the right finger movement of pushing the button.

#### Unlocked waveform

**Figure 6:**
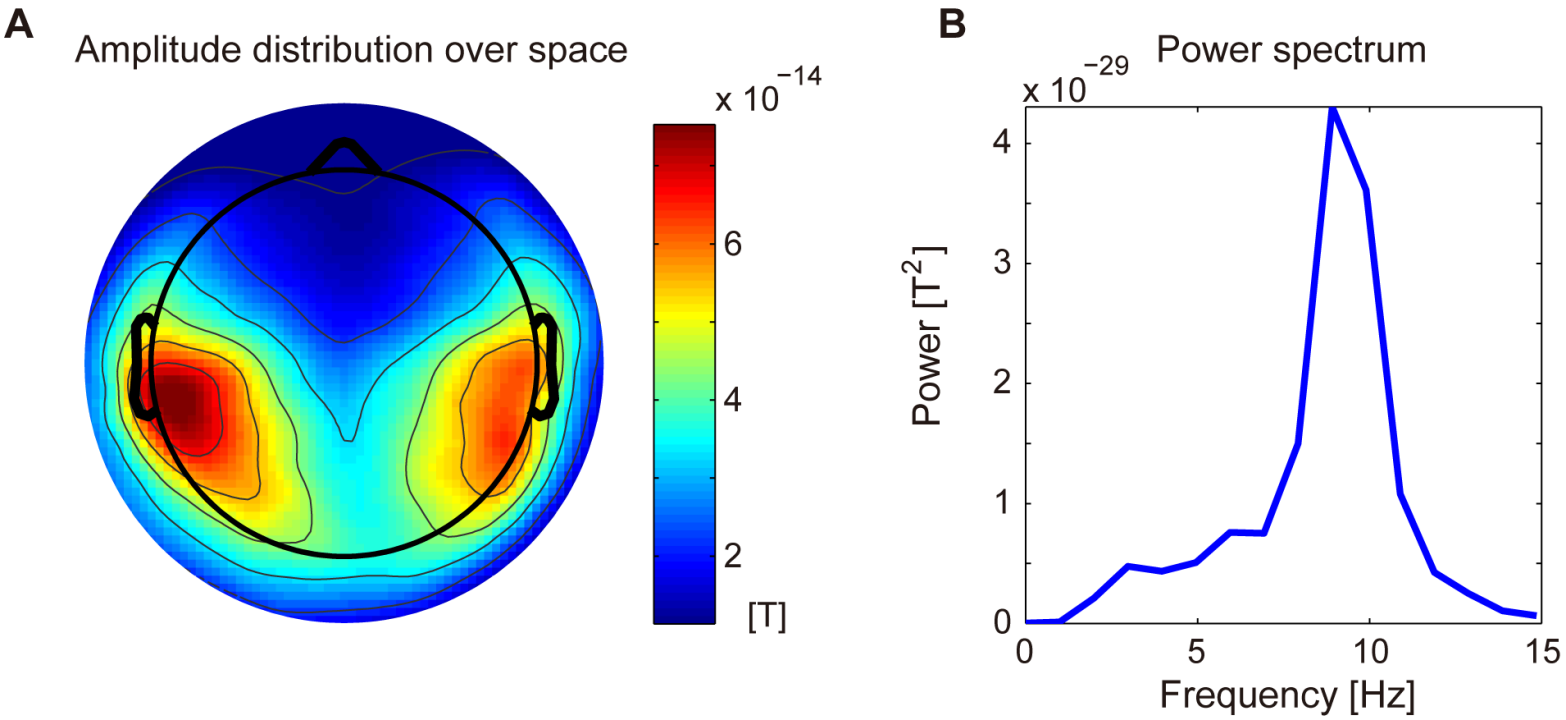
Properties of unlocked waveforms. **A**: Amplitude distribution over space of unlocked waveforms. Amplitudes are averaged across subjects. **B**: Power spectrum of unlocked waveforms. Powers are averaged across sensors and subjects.

Figure 4A shows the estimated unlocked waveform (blue lines) at the 19 axial sensors. To our knowledge, no method other than CWE can simultaneously estimate the unlocked waveform along with the stimulus- and response-locked waveforms. The unlocked waveform has large alpha oscillations at 8–10 Hz (see Figure 6B) around the temporal areas. The estimated delays of the peak in the unlocked waveform are 0.47±0.15 s. To confirm that the unlocked waveform is not an artifact generated by CWE, we compared the unlocked waveform with the estimated delay-triggered average, which was obtained by averaging the MEG epochs triggered by the estimated delays (Figure 4A, red lines). The unlocked waveform and the estimated delay-triggered average do not show clear differences (Figure 4A, right). This indicates that, at the estimated delays, there is a spatiotemporal pattern that resembles the unlocked waveform, confirming that the unlocked waveform is not an artifact.

Figure 4B shows the spatial patterns of the unlocked waveform at −0.02 s and 0.03 s after the peak of the unlocked waveform. Prominent activities are observed around the temporal areas.

Figure 4C shows the powers of the current sources estimated from the unlocked waveform. The currents have large amplitudes at the left and right superior temporal gyrus (STG) as indicated by the arrows.

#### Relation between unlocked waveform and alpha oscillations in MEG data

The unlocked waveform has large alpha oscillations at 8–10 Hz (Figure 4A). Here, we examine the relation between the unlocked waveform and alpha oscillations originally included in the MEG epochs during the Go condition.

Figures 5A and B show the amplitude distributions over space of the unlocked waveform and the alpha oscillations in the MEG epochs, respectively. At CHs 1 and 2 (Figure 5C), the alpha oscillations in the MEG epochs show large amplitudes as well as the unlocked waveform (Figure 5A and B). At CH 3 (Figure 5C), the alpha oscillations in the MEG epochs show large amplitudes, but the unlocked waveform does not have these (Figure 5A and B).

To elucidate the reasons for the similarity and the difference, we constructed time-trial images of the alpha oscillations in the MEG epochs at CHs 1–3 (Figure 5D). For CHs 1 and 2, we can observe oscillations parallel to the peak of the unlocked waveforms indicated by vertical dotted lines (black dotted lines in Figure 5D). This indicates that the alpha oscillations at CHs 1 and 2 are time-locked to the peak. In contrast, for CH 3, we cannot observe such oscillations, indicating that the alpha oscillations at CH 3 are not time-locked to the peak.

We can quantitatively confirm these observations from Figure 5E, which shows the time courses of the PLVs. The PLVs are large around the peak of the unlocked waveforms for CHs 1 and 2, but not for CH 3. This indicates that the alpha oscillations are phase-locked to the estimated delays for CHs 1 and 2 but not for CH 3.

These results show the reason why the amplitudes of the unlocked waveform are large for CHs 1 and 2 but not for CH 3. The MEG epochs include various sets of alpha oscillations synchronized across channels. The calculation of the unlocked waveform aligned one of these sets, which is dominant at CHs 1 and 2. However, it could not simultaneously align another set, which is dominant at CH 3. As a result, the alpha oscillations at CHs 1 and 2 remain, but those at CH 3 are cancelled in the unlocked waveform.

In conclusion, the unlocked waveform is considered a set of alpha oscillations synchronized across channels. The time-frequency analyses [1]–[3] cannot extract such a set of activities because it does not take into consideration the phase relation across channels.

#### Properties of unlocked waveforms

Figure 6A shows the amplitude distribution over space of the unlocked waveforms. In this figure, the amplitude distributions are averaged across the subjects. Strong amplitudes are observed around the temporal areas. The amplitude distributions of the individual subjects resemble each other (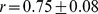, 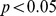, two-tailed sign test).

Figure 6B shows the power spectrum of the unlocked waveforms. In this figure, the power spectra are averaged across the sensors and subjects. Strong powers are observed at the alpha band (8–10 Hz).

The RTs of the Go condition and the estimated delays of the unlocked waveforms do not show significant correlations for most of the subjects (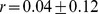, 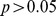 for 7 of 8 subjects).

### Role of Unlocked Waveform

Here, we show the procedure for examining the role of the estimated unlocked waveform.

The conventional procedure used to examine the roles of MEG/EEG waveforms, such as P300, is to compare the amplitudes of the waveforms across different conditions. If the amplitude is larger in one condition that needs a specific brain function, the waveforms are assumed to reflect this function. In this procedure, the compared waveforms are assumed to reflect the same brain function based on the fact that the waveforms occur with the same delay after the same event, such as stimulus onset.

In the case of unlocked waveforms, however, this assumption is not valid. Unlocked waveforms are not time-locked to observable events, so it is not clear whether unlocked waveforms estimated from different conditions reflect the same brain function. Therefore, we cannot use the above procedure.

In this study, we intended to examine the strength of the unlocked waveform’s brain activity occurring in the various conditions. To do this, we constructed a spatial filter that works as the extractor of the component reflecting the brain activity of the unlocked waveform (see the “Data Analysis” section for detailed method). By applying the spatial filter to the MEG data during the various conditions, the components reflecting the brain activity of the unlocked waveform can be extracted from the various conditions. Comparisons between the conditions were carried out by computing the powers of the extracted components, assuming that the powers indicate the strengths of the brain activities of the unlocked waveforms.

We first present our hypothesis on the role of the unlocked waveforms and then test it by using the powers.

#### Hypothesis

The unlocked waveforms have large alpha oscillations at 8–10 Hz around the temporal areas (Figures 4A and 6B). It is generally believed that the alpha oscillations reflect the inhibition of task-irrelevant activities [8]–[13]. Based on these previous studies, we hypothesized that the unlocked waveforms reflected the inhibition of the task-irrelevant activities in the temporal regions while the subject attends to the visual stimulus. Indeed, the estimated delays of the unlocked waveforms are not correlated with the RTs of the Go condition, suggesting that the unlocked waveforms are not related to the execution of the Go/NoGo task, such as decision making.

Using the powers of the components reflecting the brain activities of the unlocked waveforms, we tested the above hypothesis in the following three ways. First, to examine whether the unlocked waveforms are related to attention, we compared the powers across conditions where attention is required or not. Second, to examine whether the unlocked waveforms reflect the inhibition of the task-irrelevant activities, we examined the relation between the powers and the variability of the RTs in the Go condition. Finally, to examine whether the unlocked waveforms are specifically related to “visual” attention, we conducted an additional experiment of an “auditory” Go/NoGo task and compared the powers between the “visual” and “auditory” Go/NoGo conditions.

#### Comparison across conditions where attention is required or not

We examined whether the unlocked waveforms are related to attention. The Go and NoGo conditions require that attention be given to the stimulus, but the Passive task does not. Therefore, the hypothesis expects that the powers of the components will be large for the Go and NoGo conditions but not for the Passive task.

**Figure 7:**
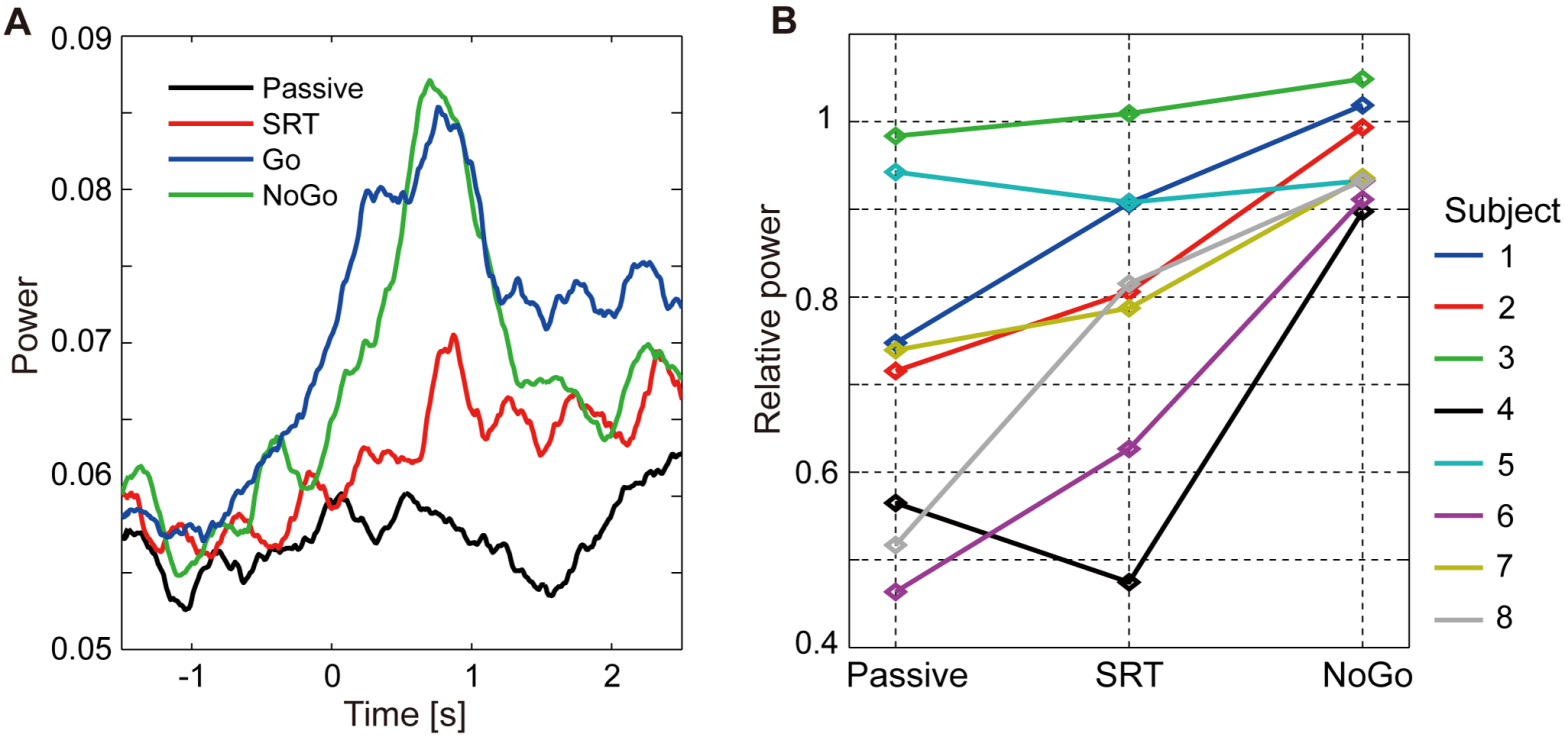
Powers of components reflecting brain activities of unlocked waveforms. **A**: Time courses of powers of components. Powers are averaged across subjects. Time 0 corresponds to stimulus onset. **B**: Relative powers of components to those of Go condition. Each line corresponds to one subject.

Figure 7A shows the time courses of the powers of the components. In this figure, the powers are averaged across the subjects. The powers clearly increase after the stimulus onset for the Go and NoGo conditions (blue and green lines) but not for the Passive task (black line).

Figure 7B shows the powers of the components relative to those of the Go condition for each subject. For the NoGo condition, the relative powers are around 1 (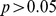, two-tailed sign test, Bonferroni-corrected), indicating that the powers of the NoGo condition are not different from those of the Go condition. For the Passive task, the relative powers are <1 for all of the subjects (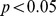, two-tailed sign test, Bonferroni-corrected), indicating that the powers of the Passive task are smaller than those of the Go condition for all subjects.

These results are consistent with our expectation, suggesting that the unlocked waveforms are related to attention.

#### Relation to variability of RTs

We examined whether the unlocked waveforms reflect the inhibition of the task-irrelevant activities. The task-irrelevant activities are considered sources of noise for the task-relevant brain process. Therefore, the inhibition of the task-irrelevant activities is believed to reduce noise. If noise is reduced, the brain process for the Go response will be less perturbed and the motor output will be stabilized, i.e. the variability of the RTs will be small. Therefore, the hypothesis expects that the variability of the RTs will be small when the powers of the unlocked waveforms are large.

**Figure 8:**
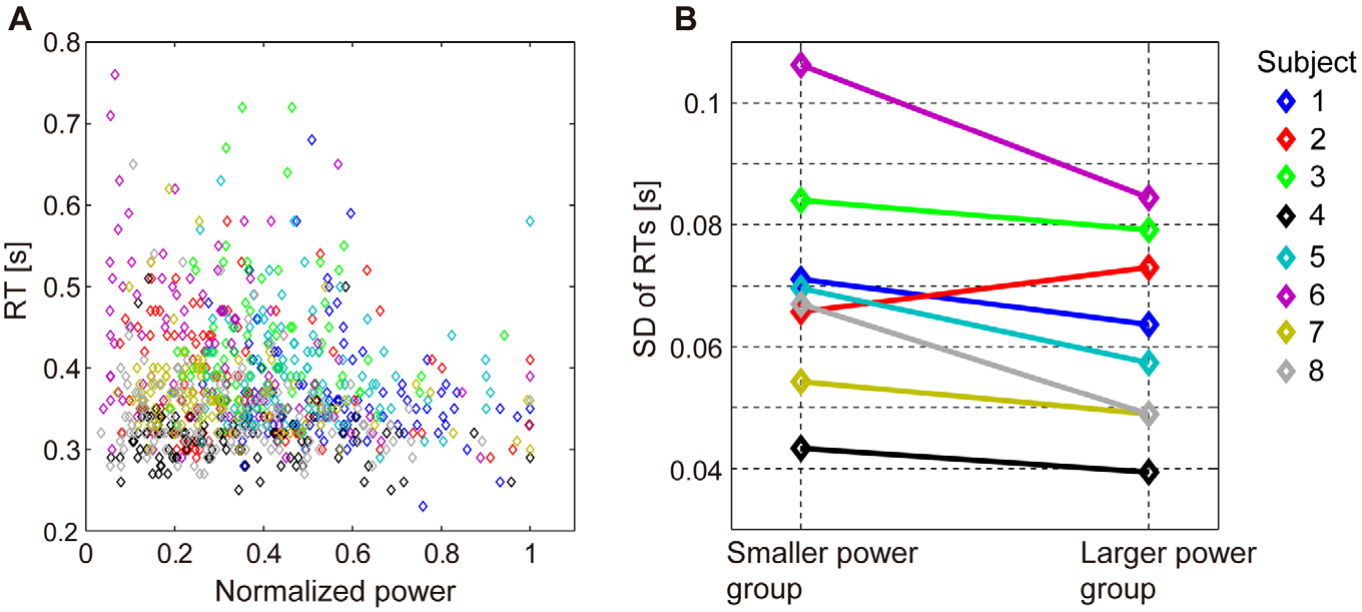
Relation between powers of components reflecting brain activities of unlocked waveforms and variability of RTs in Go condition. **A**: Scatter plot of normalized power and RTs. Each dot corresponds to one trial of one subject. **B**: SDs of RTs in smaller and larger power groups. In both figures, each color corresponds to one subject.

Figure 8A shows the scatter plot of the powers and the RTs during the Go condition. In this figure, the powers are normalized for each subject so that their maximum value becomes one. The variability of the RTs seems to be small when the powers are large. To confirm this observation quantitatively, for each subject, the trials were divided into two groups according to the normalized powers: the smaller power group and the larger power group. For 7 out of 8 subjects, the SDs of the RTs in the larger power group are smaller than those in the smaller power group (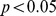, one-tailed Wilcoxon signed-ranks test) (Figure 8B). This indicates that the variability of the RTs is small when the powers are large.

This result is consistent with our expectation, suggesting that the unlocked waveforms reflect the inhibition of the task-irrelevant activities.

#### Additional experiment with auditory Go/NoGo task

We examined whether the unlocked waveforms are specifically related to “visual” attention. Parts of the task-irrelevant brain regions during visual attention, such as the auditory cortex, are task-relevant during auditory attention. Therefore, the hypothesis expects that the powers will be smaller in the case of the auditory Go and NoGo conditions.

To test this expectation, we conducted an additional experiment of an “auditory” Go/NoGo task and compared the powers between the “visual” and “auditory” conditions. In this experiment, nine healthy subjects participated, who gave written informed consent for the experimental procedures that were approved by the ATR Human Subject Review Committee. We conducted visual and auditory Go/NoGo tasks. The visual Go/NoGo task is the same as the Go/NoGo task described in the “Experimental Design” section. The auditory Go/NoGo task is the same as the visual Go/NoGo task except for the cue stimulus; an auditory cue stimulus (1500- or 2000-Hz pure tone) was presented for 1 s instead of the visual stimulus (“>” or “<”). The subjects immediately pushed the button with their right index finger after the presentation of the 2000-Hz pure tone (auditory Go) but not for the 1500-Hz pure tone (auditory NoGo). During the tasks, MEG data were recorded as described in the “Data Acquisition” section. The powers were calculated also as described in the “Data Analysis” section: the MEG data were preprocessed, unlocked waveforms were estimated from the preprocessed MEG epochs during the visual Go condition, spatial filters were prepared to extract the brain activities of the unlocked waveforms, the spatial filters were applied to the MEG epochs during the individual conditions, and the time courses of the powers were calculated from the components extracted by the spatial filters.

**Figure 9:**
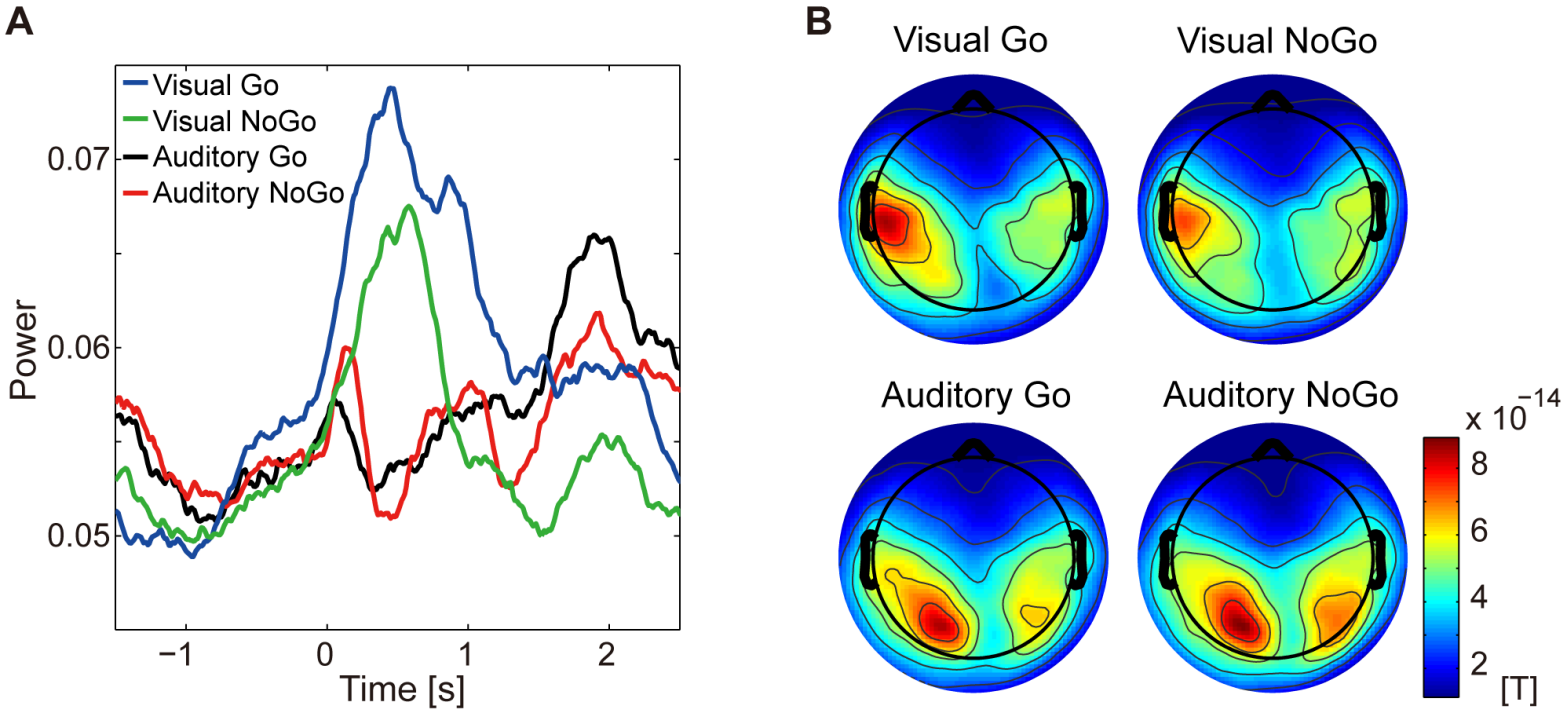
Results of additional experiment with visual and auditory Go/NoGo tasks. **A**: Time courses of powers of components reflecting brain activities of unlocked waveforms of visual Go condition. Powers are averaged across subjects. Time 0 corresponds to stimulus onset. **B**: Amplitude distributions over space of unlocked waveforms estimated from individual conditions. Amplitudes are averaged across subjects.

Figure 9A shows the time courses of the powers. In this figure, the powers are averaged across the subjects. For the visual Go and NoGo conditions (blue and green lines), the powers increase during the task period (from 0 to 1 s after the stimulus onset), which are consistent with the previous results (Figure 7A, blue and green lines). For the auditory Go and NoGo conditions (black and red lines), on the contrary, the powers show transient decreases after the stimulus onset. We averaged the powers from 0 to 1 s after the stimulus onset for each condition and subject. The averaged powers of the auditory conditions are significantly smaller than those of the visual conditions (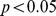, two-tailed Wilcoxon signed-ranks test).

This result is consistent with our expectation, suggesting that the unlocked waveforms of the visual Go condition are specifically related to the “visual” attention.

Further, Figure 9A indicates that the brain activities reflected by the unlocked waveforms of the visual Go condition do not occur strongly in the case of the auditory conditions. Therefore, if CWE were also applied to the MEG epochs during the auditory conditions, it is expected that unlocked waveforms with different properties would be estimated. To confirm this expectation, we estimated unlocked waveforms from all of the individual conditions, and compared their amplitude distributions over space across the conditions.

The unlocked waveforms were estimated as described in the “Data Analysis” section except that the response-locked waveforms were not assumed for the NoGo conditions. The amplitude distributions over space of the unlocked waveforms were calculated by Eq. (3) as described in the “Data Analysis” section.

Figure 9B shows the amplitude distributions over space of the unlocked waveforms. In this figure, the amplitudes are averaged across the subjects. For the visual conditions (top figures), large amplitudes are observed around the temporal areas, which are consistent with the previous results (Figure 6A). For the auditory conditions (bottom figures), in contrast, large amplitudes are observed around the occipital areas. To examine the similarity of the amplitude distributions over space between the visual Go condition and another condition, for each subject, we calculated the correlation coefficient between their amplitude distributions over space. The correlation coefficients between the visual Go and auditory conditions are significantly lower than those between the visual Go and NoGo conditions (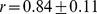 for the visual Go and auditory Go conditions, 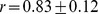 for the visual Go and auditory NoGo conditions, 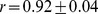 for the visual Go and visual NoGo conditions, 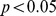, two-tailed Wilcoxon signed-ranks test). This indicates that the amplitude distributions over space of the unlocked waveforms of the auditory conditions are different from those of the visual Go condition.

This result is consistent with our expectation, supporting the results of Figure 9A.

## Discussion

In this study, we showed the usefulness of CWE for revealing cognitive unlocked brain activities. By using CWE, we successfully estimated the unlocked waveforms from the MEG data during the Go trials of the visual Go/NoGo task. The unlocked waveforms have large alpha oscillation at 8–10 Hz around the temporal areas. Based on the properties of the unlocked waveforms and the previous studies [8]–[13], we hypothesized that the unlocked waveforms reflected the inhibition of the task-irrelevant activities in the temporal regions while the subjects attend to the visual stimulus. We verified this hypothesis by comparing the powers of the components corresponding to the unlocked waveforms across the various conditions. Without using CWE, this cannot be found.

The unlocked waveform and their delays estimated by CWE were verified by comparing the unlocked waveform with the estimated delay-triggered average, which was obtained by averaging the MEG epochs triggering on the estimated delays. The similarity between them (Figure 4A) indicates that, at the estimated delays, there is a spatiotemporal pattern that resembles the unlocked waveform and that the unlocked waveform is not the artifact generated by CWE.

The amplitude distribution over space of the unlocked waveforms (Figure 5A) is different from that of the alpha oscillations in the MEG epochs during the Go condition (Figure 5B), although both have the same frequency oscillations and originate in the same data. The time-trial images and the PLVs show that the difference comes from the phase relation between channels (Figure 5D and E). Alpha oscillations have been reported as being generated by several generators, such as the visual cortex (e.g. [13]). In the MEG epochs they are mixed, while the unlocked waveforms only include oscillations synchronized across channels. Therefore, CWE successfully separated a set of alpha oscillations synchronized across channels from desynchronized ones.

Based on the previous studies on alpha oscillations [8]–[13], we hypothesized that the unlocked waveforms reflect the inhibition of the task-irrelevant activities in the temporal regions while the subject attends to the visual stimulus. We then showed four results consistent with this hypothesis. First, the delays of the unlocked waveforms are not correlated with the RTs. If the unlocked waveforms were related to the task execution, such as decision making, the delays of the unlocked waveforms would be correlated with the RTs. Second, the powers of the components corresponding to the unlocked waveforms increase during the task period for the Go and NoGo conditions but not for the Passive task (Figure 7). This result suggests that the unlocked waveforms are related to attention. Third, when the powers of the components are large, the RTs of the Go condition have small variability (Figure 8). This result suggests that the unlocked waveforms reflect the inhibition of the task-irrelevant activities. Finally, the powers of the components do not increase during the task period for the “auditory” Go and NoGo conditions (Figure 9A). This result suggests that the unlocked waveforms are specifically related to “visual” attention.

Our results and hypothesis are consistent with previous fMRI and positron emission tomography (PET) studies showing cross-modal deactivations [37]–[41]. These studies demonstrated that attention to a single sensory modality decreases activity in the cortical regions that process information from an unattended sensory modality. For example, using fMRI data and a cued detection paradigm, Mozolic et al. [40] showed the deactivation of the auditory/visual cortex during visual/auditory attention. Based on these studies, the inhibition of the brain activities in the task-irrelevant regions, such as the auditory cortex, may occur when the subject attends to the visual stimulus in the visual Go/NoGo task.

Furthermore, our results and hypothesis are consistent with previous EEG and MEG studies showing cross-modal modulation of alpha oscillations [13], [42]–[46]. These studies demonstrated that attention to a single sensory modality increases alpha oscillations in the cortical regions that process information from an unattended sensory modality. For example, using EEG data, Pfurtscheller [13] showed that there is an increase in the alpha band over the sensorimotor cortex during reading with a simultaneous decrease in the alpha band over the visual cortex, and vice versa during movement. The increased alpha oscillations were assumed to reflect the idling state of cortical networks where the transfer of specific information is reduced. Based on these studies, the increase in the alpha oscillation may occur in the task-irrelevant temporal regions when the subject attends to the visual stimulus in the visual Go/NoGo task, and CWE might extract the increased alpha oscillations as unlocked waveforms.

To our knowledge, although relations to visual attention were reported for the visual alpha (e.g. [47]) and sensorimotor mu oscillations [13], this has not been reported for the temporal alpha oscillations or so-called tau rhythms [48], [49], except in a monkey study [50]. In this study, for the first time, CWE could reveal the relation between the temporal alpha oscillations and visual attention in human subjects, although we did not expect this before applying CWE. This demonstrates the powerful ability of CWE to reveal new findings on cognitive brain functions without making any hypothesis in advance.

## Conclusions

By using CWE, we examined the unlocked waveforms in the MEG epochs during a cognitive stimulus-response task. In estimating the unlocked waveforms, we did not hypothesize their shapes or roles. After the estimation, based on the properties of the estimated waveforms, we generated a hypothesis on their roles and tested it. Consequently, CWE worked as a hypothesis generator. Generally speaking, we believe that CWE is a useful tool for data-driven and hypothesis-generating research. By applying it to MEG/EEG data during various cognitive stimulus-response tasks, we can obtain new scientific hypotheses and findings on cognitive brain functions.
